# Transcriptomics and proteomic studies reveal acaricidal mechanism of octadecanoic acid-3, 4 - tetrahydrofuran diester against *Sarcoptes scabiei* var. *cuniculi*

**DOI:** 10.1038/srep45479

**Published:** 2017-03-31

**Authors:** Xu Song, Zhenzhen Chen, Renyong Jia, Mei Cao, Yuanfeng Zou, Lixia Li, Xiaoxia Liang, Lizi Yin, Changliang He, Guizhou Yue, Zhongqiong Yin

**Affiliations:** 1 Natural Medicine Research Center, College of Veterinary Medicine, Sichuan Agricultural University, Chengdu, 611130, China; 2 Core Laboratory, Sichuan Provincial People’s Hospital, Chengdu, 610072, China; 3 College of Science, Sichuan Agricultural University, Yaan, 625014, China

## Abstract

In our previous study, a new compound, octadecanoic acid-3, 4-tetrahydrofuran diester, possessing potent acaricidal activity was obtained from neem oil. This study performed RNA-seq transcriptomics and iTRAQ proteomics to uncover the acaricidal mechanism of the compound against *Sarcoptes scabiei* var. *cuniculi.* The results of transcriptomics indicated that after treatment with octadecanoic acid-3, 4-tetrahydrofuran diester, genes related to “Energy metabolism” were significantly up-/down-regulated, including citrate cycle, oxidative phosphorylation pathway and fatty acid metabolism. Proteomics analysis showed accordant changes of proteins related to oxidative phosphorylation pathway. The target proteins of the compound were NADH dehydrogenase, Ubiquinol-cytochrome c reductase, Cytochrome c oxidase, ATP synthase, enolase and superoxide dismutase. In transcriptomics-proteomics correlation analysis, the concordance rate between protein abundances and their corresponding mRNAs was 57%, while others (43%) were discordant changes, suggesting divergent regulating effects of octadecanoic acid-3, 4-tetrahydrofuran diester. These results suggested that the acaricidal mechanism of octadecanoic acid-3, 4-tetrahydrofuran diester attributed to interference with energy metabolism, especially oxidative phosphorylation pathway.

Neem (*Azadirachta indica*), an Indian medicinal herb, is now widely planted in many Asian and African countries^1^. Neem oil, derived from the seeds or fruits of the neem tree through pressing or extraction, exhibits broad spectrum of pharmacological properties, including antiparasitic, antifungal, antibacterial, antimalarial, and anti-inflammatory activities^2^,^3^,^4^,^5^,^6^,^7^. Our previous studies obtained a new compound from neem oil, which was identified as octadecanoic acid-3, 4-tetrahydrofuran diester^8^,^9^ (Fig. 1). The acaricidal activity of the compound against *Sarcoptes scabiei* var. *cuniculi* was significantly higher than that of natural pyrethrins and abamectin, the median lethal concentration (0.082 mg/mL) is only approximately 1/33 of that of neem oil^10^,^11^. In our previous work, the activity of superoxide dismutase, peroxidase, Ca^2+^-ATPase, glutathione-s-transferases, and peroxidase of mites were significantly changed after compound treatment, prompting the hypothesis that octadecanoic acid-3, 4-tetrahydrofuran diester could regulate energy metabolism of mites^12^. However, which proteins and pathways in energy metabolism were the targets of the compound and whether the related gene expressions were regulated by the compound are still unknown.

Transcriptional profiling based on total RNA sequencing (RNA-Seq) is a powerful tool for analyzing changes of gene expression in respond to various environmental stresses^13^. Isobaric tags for relative and absolute quantification (iTRAQ) is a new protein quantification technology based on isotope labeling combined with multidimensional liquid chromatography and tandem mass spectrometry (LC-MS/MS)^14^. In this study, parallel analysis of iTRAQ-LC-MS/MS proteomics and RNA-seq transcriptomics of *S. scabiei* treated with or without octadecanoic acid-3, 4-tetrahydrofuran diester were performed for identifying changes of proteins and transcript levels for genes and revealing the acaricidal mechanism of octadecanoic acid-3, 4-tetrahydrofuran diester.

## Results

### RNA-seq transcriptomic

Illumina sequencing generated 22,473,816 clean reads. The value of Q20, a standard parameter used to assess the sequencing quality, was above 95.0% in this study, indicating the high reliability of the sequencing data. Due to the absence of reference genomic sequences, a de novo RNA-seq assembly was performed using Trinity^15^ which produced 95,306 contigs with lengths >200 bp. The transcriptome annotation showed that the *S. scabiei* unigenes did not have high similarity in the NR database and the main species distribution was in *Stegodyphus mimosarum* (22.10%). Functional characterization of the *S. scabiei* contigs was performed by assigning EggNOG annotation with BLAST+. A total of 14,123 contigs could be assigned to three functional categories: cellular processes and signaling (49.67%), information storage and processing (25.61%), metabolism (24.72%). Gene ontology (GO) was also employed to annotate the *S. scabiei* contigs. In total, 20,166 were retrieved, including biological process (37.59%), molecular function (52.36%) and cellular component (10.05%).

**Figure 2:**
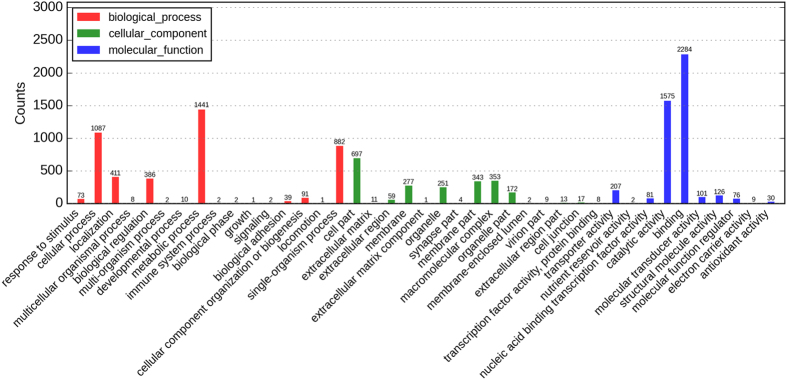
The GO annotation of differentially expressed genes.

The differentially expressed genes were identified using an R package with edgeR^16^ (q-value < 0.05). After the compound treatment, we found that 35,792 genes were significantly changed, including 10,541 down-regulated genes and 20,751 up-regulated genes. The GO annotation with BLAST2GO obtained 11,097 GO terms consisted of 40.00% “Biological process”, 40.47% “Molecular function” and 19.53% “Cellular component” (Fig. 2). The network topology of GO annotation indicated that the function of differentially expressed genes mainly distributed in several categories, such as “Glucosamine metabolism” and “Carbohydrate metabolism”. The results of KEGG pathway annotation showed that 6,366 differentially expressed genes can be enriched in 259 pathways, and 28 pathways (9 pathways in “Metabolism”) were significantly enriched (P < 0.05), including “Citrate cycle”, “Propanoate metabolism”, “biosynthesis of amino acids” and “fatty acid metabolism” (Table 1). 62 differentially expressed genes were enriched in “Oxidative phosphorylation” pathway (P > 0.05). These results suggested that octadecanoic acid-3, 4-tetrahydrofuran diester could regulate the gene expressions related to metabolism.

### Confirmation of differentially expressed genes by quantitative real-time PCR

**Figure 3:**
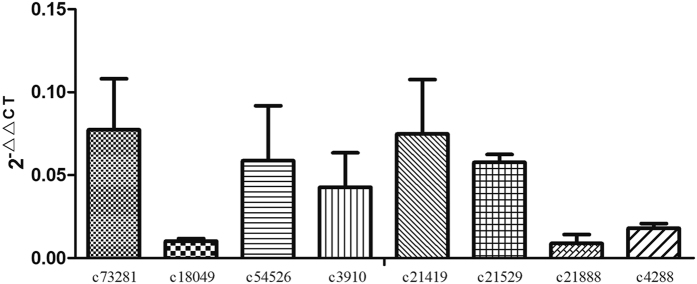
Relative expression levels of selected eight genes related to oxidative phosphorylation pathway by quantitative real-time PCR.

We used quantitative real-time PCR to validate the transcriptional pattern of randomly selected eight genes related to oxidative phosphorylation pathway in *S. scabiei*. The c73281, c18049, c54526, c21888, c3910, c21419, c21529 and c4288 unigenes were mostly involved in energy metabolism. All the genes displayed a down-regulated pattern that was in agreement with the RNA-seq analysis (Fig. 3).

### Quantitative proteomics

**Figure 4:**
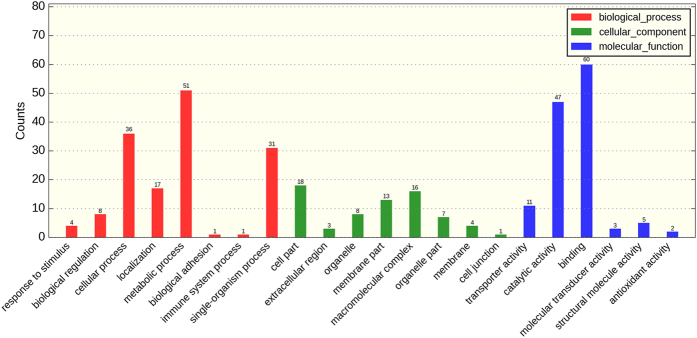
The GO annotation of differentially expressed proteins.

In order to compare the alternations of gene expression at protein level, iTRAQ proteomic analysis was performed. A total of 3,406 proteins were identified. A p-value of less than 0.05 and a 1.2-fold change of abundance were used to identify proteins that were differentially expressed between the treated group and control group. 184 differentially expressed proteins (102 up-regulated and 82 down-regulated) were detected. GO annotation obtained 149 differentially expressed proteins in biological process, 70 in cellular component and 128 in molecular function (Fig. 4). In biological process, 51 differentially expressed proteins belong to metabolic process.

KEGG pathway enrichment analysis was also performed to investigate the main effected pathways by the compound treatment. 150 differentially expressed proteins were enriched in 88 pathways (Table 2). Based on the transcriptomic data, 4 KEGG pathways were significantly regulated after treatment for 8 h, including ‘Basal transcription factors” (ko03022), “Collecting duct acid secretion” (ko04966), “Oxidative phosphorylation” (ko00190) and “Lysosome” (ko04142). In general, proteomic results demonstrated that the main acaricidal mechanism of octadecanoic acid-3, 4-tetrahydrofuran diester was to affect metabolism, especially energy metabolism.

### Correlation analysis between proteome and transcriptome

**Figure 5:**
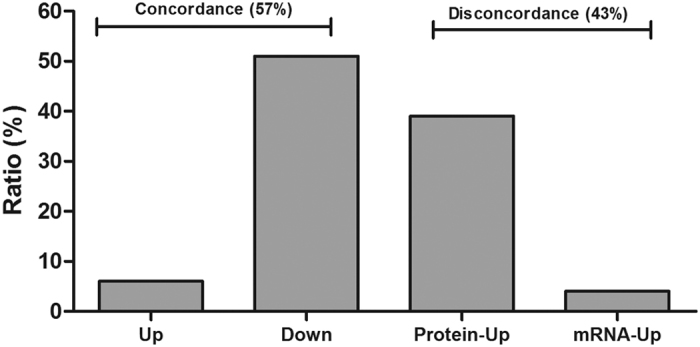
Correlation analysis between proteome and transcriptome. Up and Down represent that the differentially expressed genes and corresponding changes of protein abundances were up- and down- regulated, respectively. Protein-up represents that the protein abundances were up-regulated, but their corresponding mRNAs was down-regulated. Protein-down represents that the protein abundances were down-regulated, but their corresponding mRNAs was up-regulated.

After compound treatment, 100 differentially expressed genes and corresponding changes of protein abundance were obtained (Fig. 5). The concordance rate between protein abundances and their corresponding mRNAs was 57% (6 up-regulation, 51 down-regulation), while others (43%) were discordant changes. In energy metabolism, NADPH ubiquinone oxidoreductase (F subunit) and its mRNA were down-regulated; ATPase (E subunit) and F1-ATPase (α/β subunit) were up-regulated both in the protein and mRNA level.

## Discussion

It had been shown that there were several insecticidal ways of pesticides, mainly through interfering with insect metabolism. First, pesticides can alter the membrane structure and function causing damage of metabolic transit, such as ivermectin^17^,^18^. Second, pesticides can affect energy metabolism, such as α-Viniferin suppressing the malate dehydrogenase activity^19^. Third, pesticides can affect protein metabolism, such as chloroquinum inhibiting protein synthesis^20^. Fourth, pesticides can affect enzyme activity related to nucleotide synthesis, such as pyrazofurin inhibiting activities of lactate dehydrogenase and orotidylic acid pyrophosphorylase^21^. It has been over eight years since the new acaricidal compound, octadecanoic acid-3, 4-tetrahydrofuran diester, had been obtained from neem oil, however, we still know little about its insecticidal mechanism. Therefore, in the present study, we used RNA-seq transcriptomics and iTRAQ proteomics to identify the target of the compound.

Mitochondria, known as “the power center of the cell”, play a crucial role in cell survival, because they were major suppliers of adenosine triphosphate (ATP) which was used as a source of chemical energy. Mitochondria are the place where protein, fat and carbohydrate are oxidized mainly through tricarboxylic acid cycle and oxidative phosphorylation. Mitochondrial dysfunction could induce many diseases^22^. Therefore, mitochondrial could be the target of acaricidal drug which could produce adverse effects on mitochondrial function, finally leading to mite death. In this study, the results of RNA-seq transcriptomics showed that there were 45 differentially expressed down-regulated genes in the tricarboxylic acid cycle paths and 62 differentially expressed genes (60 down-regulated and 2 up-regulated) in oxidative phosphorylation pathway after treatment. These results suggested that octadecanoic acid-3, 4-tetrahydrofuran diester may affect the activity of related enzymes in the process of glycolysis, leading to the energy metabolic block. Besides, the differentially down-regulated genes linked to the oxidative phosphorylation included cytochrome C oxidase cbb3 subunit, cytochrome C reductase b/c1 subunit, ATP synthase and NADH dehydrogenase.

NADH-dehydrogenase, also called “Complex I”, was the first protein known as electron carriers in the transport chain, which can be oxidized by Coenzyme Q10^23^. Some pesticides, such as rotenone, could inhibit the activity of NADH-dehydrogenase^24^. Succinate dehydrogenase, also called “Complex II”, is the second protein known as electron carriers in the transport chain, which also belongs to the tricarboxylic acid cycle^25^. Our study showed that the genes of NADH-dehydrogenase and succinate dehydrogenase were down-regulated after treatment, suggesting that Octadecanoic acid-3, 4-tetrahydrofuran diester could inhibit the NADH-dehydrogenase activity and production of fumk5arate, resulting in electron transport inhibition.

Ubiquinol-cytochrome C reductase, also called “Complex III”, can catalyze coenzyme Q oxidation and reduction process of cytochrome c. Besides, the enzyme plays an irreplaceable role in donating electron from QH2 to cytochrome c receptor^26^. Cytochrome c oxidase, also called “Complex IV”, is the last protein known as electron carriers in the transport chain^26^. After treatment, the down-regulated gene expressions of Cytochrome C reductase subunit b/c1and oxidase cbb3 subunit may reduce the activity of Cytochrome C reductase and oxidase and disturb proton transport, indirectly inducing abnormal ATP synthesis.

ATP synthase, also called “Complex V”, is the terminase of oxidative phosphorylation pathway. It drives ADP and phosphate (Pi) to produce ATP by using the energy stored in the transmembrane proton gradient^27^. It is reported that after mutation of F ATP synthase subunit α, F ATP synthase could only passively transfer H^+^ and was unable to coupling with ATP, leading to losing the ability to synthesize ATP^28^. After treatment, the gene expression of F ATP synthase was up-regulated, indicating that F ATP synthase may be involved in detoxification of xenobiotics or repairing mitochondrial damage.

In accordance with the results of RNA-seq transcriptomics, iTRAQ proteomics study also showed that octadecanoic acid-3, 4-tetrahydrofuran diester could interfere with mite metabolism, especially energy metabolism. The changes of protein abundances of NADH ubiquinone oxidoreductase subunit F and ATPase linked to energy metabolism were the same as their corresponding mRNAs. Besides, enolase, one of the important glycolytic enzymes for energy metabolism, could lower host immunity for helping parasites escaping immune surveillance^29^. After treatment, the protein abundance of enolase was reduced. These results accumulated evidences for our finding that the enzymes belonging to energy metabolism were the main target of octadecanoic acid-3, 4-tetrahydrofuran diester.

In addition to these enzymes of energy metabolism, this study also found that other proteins of mites could be the target of octadecanoic acid-3, 4-tetrahydrofuran diester. Superoxide dismutase (SOD), an important protective enzyme, can eradicate the oxygen free radicals for preventing radical-related diseases^30^. In accordance with our previous studies^12^, we found that the protein abundance of SOD was down-regulated after treatment, suggesting that octadecanoic acid-3, 4-tetrahydrofuran diester had an inhibitory effect on the enzymatic activity of SOD, reducing the free radical-scavenging activity, which could eventually lead to mite death. Cathepsin, a kind of proteinase found in all animals, can degrade host proteins as its nutrients^31^. We showed that the compound could reduce the protein abundance of cathepsin, suggesting that cathepsin could be the new target of acaricidal drugs. Vitellogenin, a precursor of phospholipoprotein, is the key factor in vitellogenesis in insects^32^. It was reported that abamectin had an adverse effect on the growth and development of *haemaphysalis longicornis* and decreased ovary weight^33^. This study showed that the expression of vitellogenin was inhibited after treatment, suggesting that the compound could inhibit the development of mite ovary.

The lysosomes commonly act as waste bags to digest unwanted macromolecules in the cytoplasm, both from outside the cell and obsolete components inside the cell 34. Phagosomes in the fusion with lysosomes form phagolysosomes, which not only promote killing and degradation of microorganisms and apoptotic bodies, but also enable them to conduct autophagy to clear damaged structures^34^,^35^. In this study, transcriptomics and proteomic studies found that part of differentially expressed genes/proteins were enriched in “Phagosome” and “lysosome” pathways. The occurrence of this phenomenon may attribute to treatment with octadecanoic acid-3, 4-tetrahydrofuran diester, which could cause damages to mites leading to production of impaired macromolecules, such as proteins and organelles, finally facilitates the functions of “Phagosome” and “lysosome” pathways.

**Figure 6:**
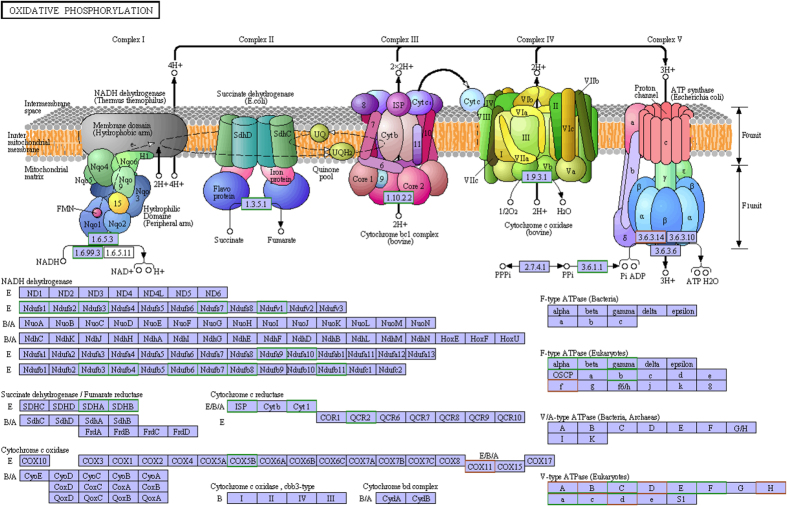
The changes of oxidative phosphorylation pathway obtained by KEGG. The subunit of each protein in green box represents down-regulation and in red box represents up-regulation.

In conclusion, the acaricidal mechanism of octadecanoic acid-3, 4-tetrahydrofuran diester mainly attributed to interference with energy metabolism, especially oxidative phosphorylation pathway (Summarized in Fig. 6). The target proteins were NADH dehydrogenase, Ubiquinol-cytochrome c reductase, Cytochrome c oxidase, ATP synthase, enolase and superoxide dismutase.

## Methods

### Treatment

*S. scabiei* larvae were isolated from infested rabbits according to the methods previously described^12^. Briefly, the octadecanoic acid-3, 4-tetrahydrofuran diester was diluted with liquid paraffin to 10 mg/mL. Then, 10 μL of solution was added into Petri dishes (8.5 mm in diameter). The mites were then placed into the solution with a needle (20 mites for each drop of the compound^8^). The liquid paraffin was served as control. All plates were incubated at 25 °C and 75% relative humidity. After treatment for 8 h, the mites were washed thrice with phosphate buffered saline (pH 7.4) and cryopreserved with liquid nitrogen for isolation of RNA and protein.

### RNA extraction, Illumina sequencing and data analysis

Total RNA was extracted from the mites using TRIzol reagent (Invitrogen) according to the manufacturer’s instructions. The concentration and quality of RNA were determined using an Agilent 2100 Bioanalyzer (Agilent, Santa Clara, CA). The construction of cDNA library and sequencing were performed as previous z6IIFFE6ed using the oligo (dT) magnetic beads. The mRNA was interrupted to short fragments after fragmentation buffer was added, followed by cDNA synthesis. The double-stranded cDNA was purified using the QiaQuick PCR Purification Kit (Qiagen, Germany) and subjected to library preparation for sequencing analysis via Illumina HiSeq^TM^ 2000.

The transcriptomic data were analyzed using a previously described protocol with modification^24^. Cleaned reads of each sample were mapped to the sequenced genome of *S. scabiei* using Bowtie^36^. Reads aligned using Bowtie were assembled into transcripts using Cufflinks, and then merged with Cuffmerge. Differential expression profiles were determined using Cuffdiff (Version: 2.1.1) with default parameters. Hierarchical clustering analysis was performed using Cluster 3.0^37^. The gene expression level is calculated by using RPKM method (Reads Per kb per Million reads)^38^.

### Quantitative real-time PCR

Quantitative real-time PCR (qRT-PCR) was employed to verify the results of RNA-Seq. Total RNA was extracted using the RNAprep pure Tissue Kit (DP431, Tiangen, China) and immediately subjected to reverse transcription using the PrimeScript™ RT reagent Kit with gDNA Eraser (RR047, TaKaRa, China). The specific primers (Table 3) for randomly selected eight genes related to oxidative phosphorylation pathway were designed using the Primer software (Version 5.0). qRT-PCR was performed at 95 °C for 30 s followed by 39 cycling of 95 °C for 5 s, 60 °C for 10 s and 72 °C for 15 s by using the iQ SYBR Green Supermix (Bio-rad, CA) on a CFX Connect™ System (Bio-rad, CA). GAPDA was used as a reference gene. The relative expression levels of target genes were analyzed using the 2^−ΔΔCT^ method^39^.

### Protein extraction and iTRAQ labeling

The mites for iTRAQ analysis were homogenized in a lysis buffer (C006225, Sangon Biotech Co., Ltd). The homogenates were centrifuged at 15,000 g for 10 min. The supernatant was purified by using 2D clean-up kit (Bio-Rad, CA). Protein concentrations and quality were determined using a Protein Assay Kit (Bio-Rad, CA).

iTRAQ analysis was carried out at Honor Tech Co. Ltd (Beijing, China) as previous reports^40^. Brief, after adjusting the pH to 8.5 with 1 M ammonium bicarbonate, total protein from each sample was reduced for 1 h at 56 °C by adding DTT to 10 mM and alkylated with 55 mM iodoacetamide for 45 min at room temperature in dark place. The protein was digested by adding trypsin (1:20, w/w). The digestion was incubated at 37 °C for overnight. A total of four samples were then labeled using iTRAQ Reagent-8plex Multiplex Kit according to the manufacturer’s instructions (Applied Biosystems, Foster City, CA,USA). Two test samples were labeled with iTRAQ tags 113 and 115, two control samples labeled with tags 117 and 119.

The labeled samples were mixed and lyophilized. They were then resuspended in 4 mL buffer containing 25% v/v acetonitrile and 25 mM NaH_2_PO_4_ (pH 2.7), and followed by fractionating using a Ultremex SCX column (4.6 × 250 mm) by HPLC system (Shimadzu LC-20AB). A total of twenty fractions obtained were desalted using a Strata X C18 column (Phenomenex, USA) and then vacuum-dried.

### Liquid chromatography-mass spectrometry

Each of the dried fractions was dissolved with buffer containing 5% v/v acetonitrile and 0.1% Formic acid and centrifuged at 20,000 g for 10 min. The peptides were separated by a 2 cm C18 trap column (inner diameter 200 μm) on an nano HPLC (Shimadzu LC-20AD). The eluted peptides were analyzed using nanoelectrospray ionization, followed by tandem mass spectrometry (MS/MS) in an Q-Exactive (Thermo Fisher Scientific, San Jose, USA) coupled with an online HPLC system. Intact peptides were detected in the Orbitrap with a resolution of 70,000. Peptides were selected for MS/MS using higher energy collision dissociation (HCD) operating mode with a normalized collision energy setting of 27%. A data-dependent procedure was applied for the three most abundant precursor ions above a threshold ion count of 20,000 in the MS survey scan.

### Proteomic data analysis

The MS spectra were analyzed by a thorough search using Mascot software (version 2.3.02, Matrix Science Inc, Boston, MA) against *S. scabiei* database. To reduce false positive results, all data were reported based on a 95% confidence and false discovery rate (FDR) less than 1%. For quantitative analysis, a protein must have at minimum one unique peptide matches with iTRAQ ratios. A 1.2-fold cutoff value was used to identify up-regulated and down-regulated proteins with a p-value of less than 0.05.

### Functional annotation

Interproscan 4.5 was used to identify conserved domains from peptide translations and database for protein sequences matching to MS peptides^41^. Gene ontology (GO) annotations for identified proteins based on BLAST results were performed using Blast2GO (http://www.blast2go.de/). Sequences were assigned to KEGG orthologous groups by comparison with the KEGG protein database^42^,^43^,^44^. Custom perl scripts were used to bin orthologous groups into broad categories and KEGG modules.

## Additional Information

**How to cite this article:** Song, X. *et al*. Transcriptomics and proteomic studies reveal acaricidal mechanism of octadecanoic acid-3, 4 - tetrahydrofuran diester against *Sarcoptes scabiei* var. *cuniculi. Sci. Rep.* **7**, 45479; doi: 10.1038/srep45479 (2017).

**Publisher's note:** Springer Nature remains neutral with regard to jurisdictional claims in published maps and institutional affiliations.

## Figures and Tables

**Figure 1:**
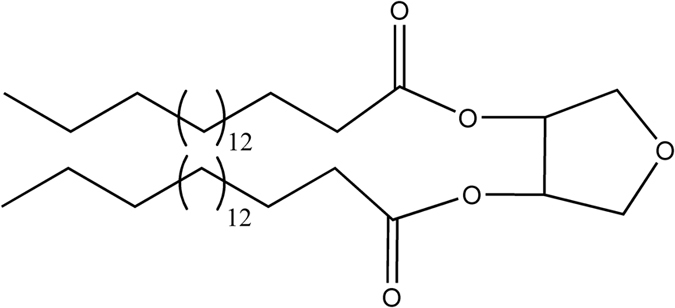
The structure of octadecanoic acid-3, 4 - tetrahydrofuran diester.

**Table 1:** KEGG pathway annotation of differentially expressed genes.

Pathway	DEGs with pathway annotation(6366)	P-value
Phagosome	85(1.34%)	1.44E-06
Lysosome	112(1.76%)	1.57E-06
Antigen processing and presentation	48(0.75%)	1.92E-05
Thyroid hormone synthesis	58(0.91%)	0.000133610
Protein digestion and absorption	18(0.28%)	0.000663957
NOD-like receptor signaling pathway	21(0.33%)	0.000812845
ECM-receptor interaction	28(0.44%)	0.001944168
Proximal tubule bicarbonate reclamation	11(0.17%)	0.005283026
Carbon fixation in photosynthetic organisms	27(0.42%)	0.008627603
PI3K-Akt signaling pathway	100(1.57%)	0.009775528
Estrogen signaling pathway	70(1.10%)	0.010183374
Focal adhesion	70(1.10%)	0.013006902
Carbon metabolism	124(1.95%)	0.016390223
Synaptic vesicle cycle	30(0.47%)	0.016564238
Fatty acid degradation	50(0.79%)	0.01661521
Alanine, aspartate and glutamate metabolism	31(0.49%)	0.017463452
Plant-pathogen interaction	20(0.31%)	0.019002489
Collecting duct acid secretion	16(0.25%)	0.020214136
Hematopoietic cell lineage	7(0.11%)	0.021354465
Biosynthesis of amino acids	81(1.27%)	0.025924987
Fatty acid metabolism	67(1.05%)	0.031310858
Fat digestion and absorption	15(0.24%)	0.031332109
Propanoate metabolism	30(0.47%)	0.034159996
Mineral absorption	6(0.09%)	0.041050613
Tight junction	42(0.66%)	0.043123628
Hippo signaling pathway -fly	43(0.68%)	0.043563262
Citrate cycle (TCA cycle)	45(0.71%)	0.044335969
Retinol metabolism	32(0.50%)	0.049045750
Oxidative phosphorylation	62(0.97%)	0.172722237

**Table 2:** KEGG Pathway analysis of differentially expressed proteins.

Pathway	Diff Proteins with pathway annotation(150)	Pathway ID
Basal transcription factors	2(1.33%)	ko03022
Collecting duct acid secretion	3(2.00%)	ko04966
Oxidative phosphorylation	7(4.67%)	ko00190
Lysosome	6(4.00%)	ko04142
RIG-I-like receptor signaling pathway	1(0.67%)	ko04622
Circadian rhythm - fly	1(0.67%)	ko04711
Synaptic vesicle cycle	3(2.00%)	ko04721
Propanoate metabolism	3(2.00%)	ko00640
Aminobenzoate degradation	1(0.67%)	ko00627
Glycosphingolipid biosynthesis - ganglio series	1(0.67%)	ko00604
Glycosylphosphatidylinositol(GPI)-anchor biosynthesis	1(0.67%)	ko00563
Base excision repair	1(0.67%)	ko03410
Styrene degradation	1(0.67%)	ko00643
DNA replication	1(0.67%)	ko03030
MAPK signaling pathway - yeast	1(0.67%)	ko04011

**Table 3:** The primers for quantitative real-time PCR.

Gene	Forward Primer (5′-3′)	Reverse Primer (5′-3′)
GAPDH	TGCCGTGGGTGGAATCATAC	CGAGACACGATGGTGAAGGT
c73281	GCGTACATAAATACTCGG	GGGCATTATCATTAGCTC
c18049	GCTTTGAATCTTGAGACGGACA	GCCCAGAGCATCTACGAC
c54526	GGCCAACCAATTAATCCAG	TTCATTATGTGGCAAACCAG
c21888	TAGACTTCCGACTTTTACGC	TTGATGGAATCCAACGACC
c3910	TTCGGATCAATTTCGGTG	CATTTGAGAAAACGCCTA
c21419	TTGGATAATGCCATTGCCT	TCGCTAATGCCACAATATCCAG
c21529	AGGTTGTAAAATGACCGAA	TGCACTGATTCAATATCGAAC
c4288	TACTTATTCCAGCCATTTTCGTT	TTCTATCGCTTCATGTTAGGTCT
